# Letter to the Editor

**Published:** 2010-06

**Authors:** Salvatore Lentini, Filippo Benedetto, Amedeo Carmignani, Roberto Gaeta

**Affiliations:** Cardiovascular and Thoracic Department, Policlinico G Martino, University of Messina, Messina, Italy; Cardiovascular and Thoracic Department, Policlinico G Martino, University of Messina, Messina, Italy; Cardiovascular and Thoracic Department, Policlinico G Martino, University of Messina, Messina, Italy; Cardiovascular and Thoracic Department, Policlinico G Martino, University of Messina, Messina, Italy; Cardiac Surgery Unit, Ospedale San Carlo, Potenza, Italy

Dear Sir,

We read with interest the recent paper from Prof Suzer and co-workers on concomitant coronary artery bypass grafting and descending aorta-to-bifemoral artery bypass via sternotomy.^1^ The authors are to be commended for their work.

The concept of revascularising both the coronary tree and the lower limb within the same surgical session is not new, and has been used by different surgical groups both previously^2^,^3^ and recently.^4^

In the past we have also used concomitant coronary and lower-limb surgical revascularisation. We normally utilised the ascending aorta as inflow for the bifurcated vascular prosthesis to the femoral arteries. The prosthesis, usually a Dacron tube, was passed in front of the heart, behind the sternum, usually protected inside the pericardial space, and then through the anterior abdominal wall to reach the groin. The indication for this type of concomitant surgery was usually when there was risk of lower-limb ischaemia during postoperative coronary surgery, or the presence of critical peripheral arterial ischaemia in an unstable cardiac patient. The refined technique described by the authors represents an advancement of the previously used technique where the tube was passed in front of the heart.

From our past experience we would like to add a few points on this subject. We consider that there is theoretically an increased risk when more surgery is performed in the same session. In particular, a marked reduction of peripheral vascular resistance may occur due to an abrupt increase of the vascular bed following lower-limb revascularisation. This haemodynamic condition would happen just after discontinuation of cardiopulmonary bypass, and therefore at a critical moment for the heart. This status could necessitate increased dosage of vasopressor agents with the risk of postoperative peripheral vasoconstriction, or arterial coronary graft spasm.

There is an increased risk because one is treating in the same surgical session not only the mediastinum, but also the retroperitoneum and peripheral arterial vessels (femoral). In particular, the prosthesis passage through the retroperitoneal space or the abdominal wall may represent a source of occult bleeding in the postoperative period.

There is also a risk of prosthesis infection, as mentioned by the authors, both in short- and long-term follow up. The technique proposed by Suzer *et al.* would leave the prosthesis confined in the posterior mediastinum, and not in the anterior one, just in contact with the heart. This could be considered an improvement compared to the technique using the ascending aorta as inflow, where an infection could propagate through the Dacron tube from the groin directly to the pericardial space. However, even in the technique described by our colleagues, we believe some risk of infection propagation from one site to another still persists. In the past, we observed a patient where infection propagated from the groin up to the pericardial space, this happening a few years after surgery.

Some patients may need revascularisation below the femoral artery, such as patient number 3 in the Suzer *et al.* report. Actually, in our series of patients, this represented an adjunctive risk, especially with concomitant bilateral revascularisation below the knee. This was probably due to extensive peripheral disease, advanced age and the presence of concomitant pathology in this subgroup of patients. Postoperative exacerbation or abrupt onset of peripheral ischaemia in those patients may represent a significant complication.

Now, considering that we have a patient with both coronary and peripheral vascular disease, the question is: ‘In the recent era of endovascular treatments (both coronary and peripheral vascular), do we still need concomitant surgery?

Currently, whenever possible, we usually prefer a staged approach, treating in the first instance the more critical region (coronary or peripheral). We then use endovascular options as a bridge to a more definitive treatment in one of the two regions to allow surgery on the other. We believe that concomitant coronary and lower-limb surgery is feasible, however it should be reserved for very selected cases, and the possible complications should be weighed up.

We again congratulate Prof Suzer and co-workers for their interesting work.
